# A Quantitative Examination and Comparison of the Ability of Australian Gentamicin Dosing Guidelines to Achieve Target Therapeutic Concentrations in Neonates

**DOI:** 10.3390/antibiotics14010048

**Published:** 2025-01-08

**Authors:** Luke E. Grzeskowiak, Sheree Wynne, Michael J. Stark

**Affiliations:** 1 College of Medicine and Public Health, Flinders University, Adelaide 5042, Australia; 2 Adelaide Medical School, Robinson Research Institute, The University of Adelaide, Adelaide 5005, Australia; michael.stark@adelaide.edu.au; 3 Women and Kids Theme, South Australian Health and Medical Research Institute, Adelaide 5000, Australia; 4 SA Pharmacy, Flinders Medical Centre, SA Health, Adelaide 5042, Australia; sheree.wynne@sa.gov.au; 5 Department of Neonatal Medicine, Women’s and Children’s Hospital, Adelaide 5006, Australia

**Keywords:** gentamicin, infant, newborn, premature, pharmacology, pharmacokinetics, sepsis

## Abstract

**Background:** Effective gentamicin dosing is crucial to the survival of neonates with suspected sepsis but requires a careful balance between attaining both effective peak and safe trough concentrations. We aimed to systematically compare existing gentamicin dosing guidelines for neonates in Australia to determine the extent to which they reach therapeutic targets. **Methods:** Simulations of a single gentamicin dose to a virtual representative neonatal population according to each Australian guideline were performed using population pharmacokinetic modelling. We determined the proportion of neonates who would achieve peak gentamicin concentrations of ≥5 or ≥10 mg/L and trough concentrations of ≤1 or ≤2 mg/L. We calculated the probability of target attainment (PTA) according to gestation at birth (22 to 40 weeks) and postnatal age (1–7, 8–14, 15–21, 22–28 days). **Results:** Five unique dosing guidelines were identified. Guidelines varied considerably with respect to dose (4.5 to 7 mg/kg), dosing interval (24 to 48 h), and characteristics used to individualise dosing regimens (e.g., gestation at birth and postnatal age). Guidelines were satisfactory in routinely achieving effective peak concentrations ≥ 5 mg/L, but PTAs for effective peak concentrations ≥ 10 mg/L varied considerably from 5% to 100% based on dose, gestation, and postnatal age. PTAs for trough concentrations ≤ 1 mg/L ranged from 0% to 100%, being lowest among extremely preterm infants. **Conclusions:** Current Australian gentamicin guidelines demonstrate significant variability in their ability to achieve defined therapeutic targets, necessitating efforts to improve standardisation of dosing recommendations. Further research to define optimal pharmacodynamic targets in neonates with respect to clinical outcomes are also urgently warranted.

## 1. Introduction

Neonatal sepsis is a significant cause of morbidity and mortality worldwide. A recent meta-analysis reported a global prevalence of 2824 neonatal sepsis cases per 100,000 live births and corresponding mortality rate of 17.6% [1]. Neonatal risk factors for sepsis mortality include prematurity, lower postnatal age and immaturity of the immune system [2,3]. Neonatal sepsis is divided into early- and late-onset forms. These differ by mode of acquisition as well as timing of onset. Early-onset sepsis (EOS) usually results from pathogens acquired intrapartum, through vertical transmission from the mother. In contrast, late-onset sepsis (LOS) is usually acquired from the environment. The time point separating EOS from LOS remains the subject of debate and ranges from 48 h to seven days [4]. The clinical presentation of EOS and LOS are similar, characterised often by nonspecific signs and symptoms. Common early signs include fever, tachypnoea, lethargy, and poor feeding, which if left untreated, can rapidly progress to severe illness with vital sign instability, central nervous system manifestations such as irritability, lethargy, or seizures, and, ultimately, multi-organ system dysfunction and failure [5].

In the United Kingdom, the most common pathogens causing early-onset neonatal sepsis are Group B *Streptococcus* (50%) and *Escherichia coli*. (18%) [6]. This is in line with Australia, where Group B *Streptococcus* and *Escherichia coli*. combined account for 57% and 68% of organisms causing early-onset sepsis in infants born at 23–36 weeks gestation and greater than 36 weeks gestation, respectively [7]. More recent data demonstrate that *Escherichia coli.* is now the dominant microorganism of very preterm early-onset sepsis in Australia and New Zealand [8]. While different pathogens are often isolated in cases of EOS compared with LOS, reflective of their different modes of acquisition, considerable overlap remains. In the case of LOS, common pathogens include Coagulase-negative staphylococci, *Staphylococcus aureus*, and *Enterococcus* spp. [5].

In Australia, gentamicin is the most frequently prescribed antibiotic for suspected neonatal sepsis, accounting for 43.1% of all prescriptions [9]. In most cases, gentamicin is prescribed in combination with a beta-lactam antibiotic such as benzylpenicillin [9]. Gentamicin displays concentration-dependent killing both in vitro and in vivo, and a high ratio between the peak plasma concentration and the minimum inhibitory concentration (MIC) of the infecting microorganism is considered important for a therapeutic response [10,11]. Studies suggest that optimal gentamicin efficacy is achieved with a plasma peak concentration over an MIC ratio (Cmax/MIC) of 8 to 10. However, achieving efficacy targets must be balanced against the potential risk of ototoxicity and nephrotoxicity related to high trough serum concentrations. While nephrotoxicity is typically transient, ototoxicity may be permanent. In order to minimise potential toxic effects, it has been suggested that trough concentrations should not exceed 1 to 2 mg/L [12]. Therefore, achieving therapeutic targets requires careful rationalisation of the dosing recommendations [12].

Significant variability in gentamicin pharmacokinetics is evident in the neonatal population, reflecting rapid changes in postnatal kidney function and body composition influencing its clearance and volume of distribution. Gentamicin clearance is strongly correlated with renal function and is almost entirely eliminated by the kidneys [12,13]. Renal function is highly dependent on gestational age (GA) (with nephrogenesis not completed until after 34 to 35 weeks gestation) and changes rapidly in the first week of life following birth, owing to increased renal and intrarenal blood flow [14]. Thus, renal elimination of gentamicin in neonates is largely linked to both GA and postnatal age (PNA). In comparison, gentamicin distribution is largely limited to the extracellular fluid compartment. As gestational age is inversely correlated with body water content [15], higher doses per kilogram are required at earlier gestational ages to achieve effective peak concentrations.

Despite improved pharmacokinetic understanding of gentamicin in neonates, significant differences in dosing recommendations are evident locally and internationally. Pharmacometric analyses provide an opportunity to evaluate different dosing regimens with respect to target attainment and provide a quantitative approach towards the development of improved treatment recommendations in neonates. Therefore, the aims of this study were to (i) assess the variability in empiric dosing of gentamicin in Australian neonatal intensive care units (NICUs) and (ii) evaluate and compare the probability of target attainment of dosing regimens with respect to therapeutic efficacy and safety targets.

## 2. Results

### 2.1. Comparison of Neonatal Gentamicin Dosing Guidelines

Across the 23 Australian NICUs, five unique guidelines were identified (Table 1). Significant variability was evident across these guidelines according to dose (4.5 to 7 mg/kg), dosing interval (24 to 48 h), and patient characteristics used to individualise dosing regimens (i.e., GA, PNA, and postmenstrual age (PMA)). Cut-off values to define subgroups often differed across guidelines. Only two guidelines recommended a target peak concentration, one of 5–12 mg/L and the other > 10 mg/L. Most (4 of 5) guidelines recommended a target trough concentration of ≤1 mg/L. Notably, no one neonate with the same characteristics would receive the same dosing regimen across all five guidelines.

### 2.2. Population Pharmacokinetic Model and Simulation Dataset

A total of 10 potentially suitable models were identified in the literature that described the population pharmacokinetics of gentamicin in neonates [13,16,17,18,19,20,21]. The model reported by Fuchs et al. was selected as the most appropriate [16]. The selected model was built upon a large cohort of neonates (994 preterm and 455 term newborns) including a total of 3039 gentamicin concentrations. The model provided appropriate representation of the target population with respect to GA, PNA, and body weight. The model describes gentamicin pharmacokinetics according to a two-compartmental model, with first-order elimination from the central compartment. Body weight, GA, PNA, and dopamine co-administration were included as covariates influencing gentamicin pharmacokinetics.

The in silico neonatal population comprised of 36,812 neonates, with approximately equal representation across each GA and PNA group. The distribution of, and correlation between, patient GA, PNA, birth weight, and current weight are illustrated in Figure S1. Given no data on dopamine administration were available for the in silico neonatal population, all simulations assumed no dopamine co-administration. Notably, dopamine co-administration only has a marginal effect on gentamicin clearance (12% reduction) [16].

### 2.3. Peak Concentrations

The median PTA for peak concentrations ≥ 10 mg/L ranged from 24% to 100% across guidelines (Table 2).

**Figure 1:**
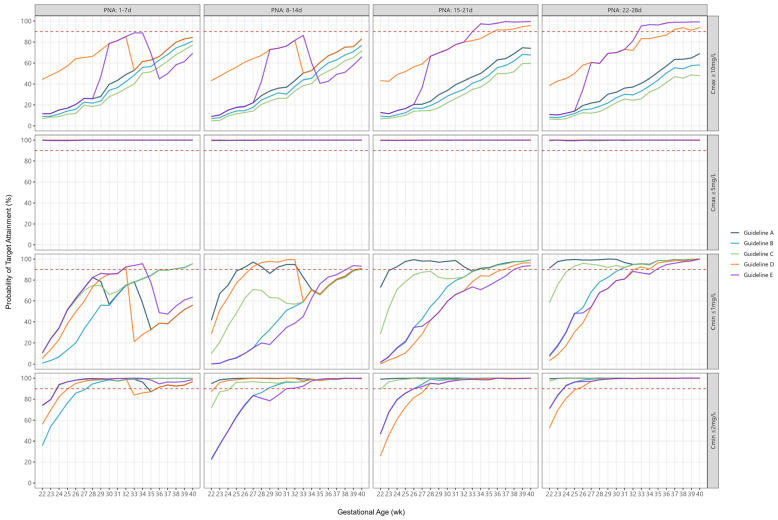
Probability of target attainment for peak and trough concentrations according to individual guidelines, by gestational age and postnatal age. The dashed line represents 90% probability of target attainment.

PTA typically gradually increased in accordance with advancing GA at birth (Figure 1). In contrast, PTA for peak concentrations ≥ 5 mg/L were consistently >99% across all guidelines (Table 2) and all GAs (Figure 1).

Increasing GA was associated with higher median peak concentrations, with some guidelines achieving median values as high as 15 mg/L according to GA and PNA subgroups (Figure S2).

**Figure 2:**
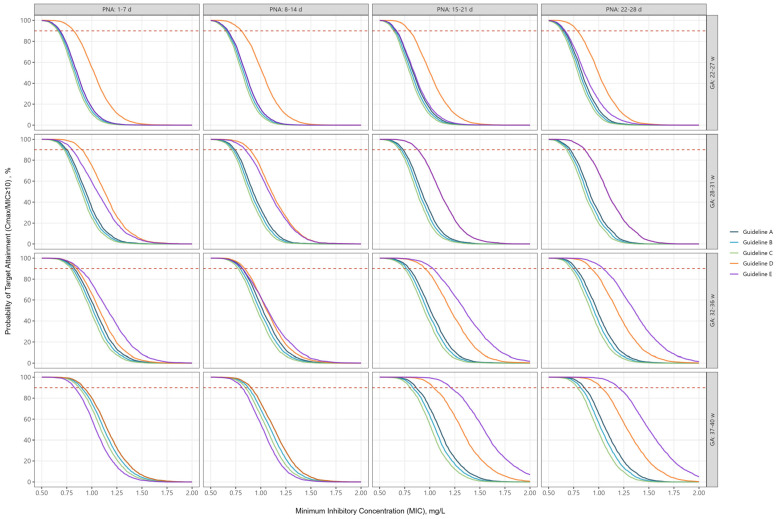
Probability of target attainment for peak ≥10× minimum inhibitory concentration according to individual guidelines, by gestational age and postnatal age. The dashed line represents 90% probability of target attainment.

When assessed, according to PTA of peak/MIC ratio ≥ 10, most guidelines were only able to achieve a PTA > 90% for an MIC of 0.6 among extremely preterm infants (Figure 2). In contrast, among term births, a PTA > 90% was achieved for MICs ranging from 0.8 to 1.2.

### 2.4. Trough Concentrations

The median PTA ranged from 35% to 100% for trough concentrations ≤ 1 mg/L and 90% to 100% for trough concentrations ≤ 2 mg/L across guidelines (Table 2). When assessed according to individual GA and PNA subgroups, PTA was consistently lowest for extremely preterm infants, irrespective of postnatal age (Figure 1). For most guidelines a significant decrease in PTA was evident around GA of 33–35 weeks within the first two weeks of life, corresponding to shortening of the dosing intervals (Figure 1). The relationship between increasing GA and median trough concentrations was less consistent, largely owing to frequent changes in dose and/or dosing frequency (Figure S3).

### 2.5. Combined Peak and Trough Concentrations

**Figure 3:**
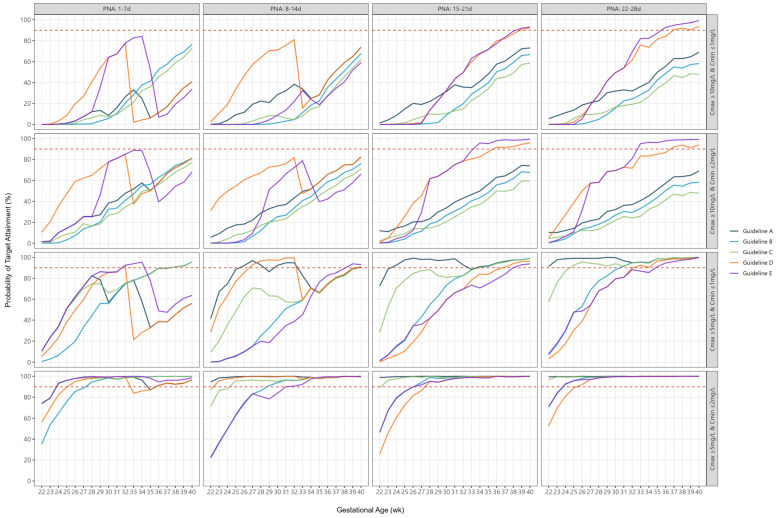
Probability of target attainment for combined peak and trough target concentrations according to individual guidelines, by gestational age and postnatal age. The dashed line represents 90% probability of target attainment.

PTAs for combined peak and trough targets were significantly lower than PTAs for individual targets. The median PTA ranged from 3% to 54% for combined peak concentrations ≥ 10 mg/L and trough concentrations ≤ 1 mg/L, increasing to 23% to 75% for combined peak concentrations ≥ 10 mg/L and trough concentrations ≤ 2 mg/L (Table 2). In contrast, the median PTA ranged from 35% to 99% for combined peak concentrations ≥ 5 mg/L and trough concentrations < 1 mg/L, increasing to 90% to 100% for combined peak concentrations ≥ 5 mg/L and trough concentrations < 2 mg/L (Table 2). Targeting a lower peak and higher trough concentration resulted in greater than 90% PTA across most GAs, except for extremely preterm infants (Figure 3). In contrast, targeting higher peak and/or lower trough concentrations led to low PTAs across almost all GAs (Figure 3).

## 3. Discussion

Our findings demonstrate significant variability in the ability of existing gentamicin guidelines used across Australian neonatal units to reach desired therapeutic targets. Critically, all fail to consistently achieve effective peak and safe trough targets across all neonatal subpopulations, with extremely preterm infants particularly vulnerable to possible toxicity from high trough concentrations and reduced efficacy due to lower peak concentrations.

A major challenge with respect to optimising gentamicin dosing lies in the fact that evidence surrounding optimal pharmacodynamic targets specific to the neonatal population is lacking and largely relies on extrapolation from adults. With respect to peak target concentrations, most guidelines failed to achieve adequate PTA for peak concentrations ≥ 10 mg/L, whereas all achieved peak concentrations ≥ 5 mg/L. It is suggested that the optimal exposure target for efficacy should be a Cmax/MIC ≥ 10, as this has been previously associated with improved efficacy, albeit not in a neonatal population [12]. Therefore, current peak concentrations of ≥10 mg/L and ≥5 mg/L correspond to MICs of 1 mg/L and 0.5 mg/L, respectively. Given a recent evaluation of Gram-negative pathogens causing neonatal infections demonstrated that increasing gentamicin minimum inhibitory concentrations (MICs), even in the susceptible range, is associated with increased mortality [6], the inability for many Australian guidelines to achieve higher peak concentrations is a concern. Ideally, dosing strategies should rely on knowledge of individual MICs for pathogens causing neonatal sepsis, but the majority of gentamicin treatment is done empirically, without a proven cause of sepsis being known. Therefore, treatment targets should be set based on the most likely and virulent pathogens causing neonatal sepsis. Using such approaches, targeting MICs > 0.75 mg/L appear justified, but this can only be reliably attained by increasing doses above 5 mg/kg.

Balanced against the need to attain adequate peak concentrations is the desire to achieve low trough concentrations. While most Australian gentamicin guidelines recommend a desired therapeutic trough target ≤ 1 mg/L, our analysis demonstrates that this is difficult to obtain for most neonates. Accepting a higher trough concentration ≤ 2 mg/L greatly increases PTAs across all neonates except for the cohort of extremely preterm infants. There remains ongoing debate as to what the optimal target trough concentration should be with respect to minimising potential risks of nephrotoxicity and ototoxicity, but findings from this quantitative examination highlight the need for guidelines to either revise their dosing recommendations or target trough concentrations to better align with expected PTA.

The significant variation in neonatal gentamicin dosing recommendations is not unique to Australia, with international studies also demonstrating significant differences across different sites [22]. Guideline variations include differences in dose and dosing interval, as well as patient characteristics used for dose individualisation (i.e., GA, PNA, and/or PMA). More precise dose individualisation may enhance target attainment across different patient characteristics. However, this approach comes at the cost of more complex dosing recommendations, increasing the risk for prescription errors, a common problem in neonatal clinical practice [23]. Therefore, many guidelines opt for dosing harmonisation (i.e., same dose for all but with variation in dosing intervals) to simplify prescribing. All guidelines had similar effectiveness in their ability to achieve peak concentrations ≥ 5 mg/L. Those guidelines opting for more complex dosing recommendations, however, were more likely to achieve peak concentrations of ≥10 mg/L among certain subgroups.

Few studies have robustly assessed the ability of contemporary pharmacological guidelines to meet therapeutic targets [20,22]. Van Donge (2018) identified and compared multiple international and Swiss NICU guidelines using a model-based simulation approach on a real European neonatal population treated for suspected infection [22]. PTAs were measured for the entire population and found that the guidelines attained effective peak concentrations of ≥5 mg/L, and safe trough concentrations of ≤2 mg/mL, in >90% of the population. This study did not however explore the PTA across individual GA and PNAs. Furthermore, the evaluations were performed using a real population which was not uniformly distributed, potentially resulting in some age groups being underrepresented in the population. Such analyses are heavily weighted towards infants of higher GA, masking inadequacies of PTAs at lower gestations. In contrast, Bijleveld et al. developed a model from preterm neonatal data from a Dutch NICU and performed simulations for 1000 virtual patients with GAs of 25, 30, 32, 37, and 40 weeks and corresponding body weights of 830, 1500, 2000, 2500, and 3500 g, respectively, to evaluate their current dosing guidelines [20]. Their guidelines were found to be inadequate for most GAs, as the median concentrations were not within their peak target of 8–12 mg/L or below their trough target of 1 mg/L. While this study explored a range of GAs, it was limited by the small population size and the static body weight assigned to each GA.

Beyond improved dosing recommendations, efforts to improve the safety of gentamicin use in neonates need also consider evidence and approaches to support the more rational use of antibiotic therapy and reduce treatment duration. For example, significant efforts have been made in the development and implementation of a neonatal sepsis calculator to better predict the likelihood of EOS in infants born ≥34 weeks gestation. There is moderate quality evidence that the use of a sepsis calculator is associated with substantial reductions in antibiotic use (including gentamicin), with no corresponding increase in mortality or neonatal unit readmissions [24]. In the setting of culture-proven sepsis, gentamicin is often switched to more targeted therapy. Where sepsis is suspected or confirmed, gentamicin should be used for the shortest possible duration. In many cases where treatment is initiated based solely on risk factors, but the neonate remains asymptomatic and blood cultures remain negative at 36–48 h, treatment should be discontinued [25]. In the setting of culture-proven sepsis, there is emerging evidence in support of shorter treatment durations for uncomplicated neonatal septicemia [26,27,28], but overall evidence regarding optimal treatment durations remain uncertain.

The major strength of the current study lies in the application of a robust population pharmacokinetic model to a virtual representative neonatal population with a uniform age distribution and range of correlated body weights, to ensure all age groups were fairly and accurately represented and evaluated.to simulate PTAs, as well as the use of multiple therapeutic targets. This study has a number of limitations. Firstly, there is a lack of universally agreed pharmacokinetic/pharmacodynamic targets, prohibiting the ability to define what the optimal dosing regimen should be across GA and PNA groupings. Further research examining the relationship between pharmacokinetic/pharmacodynamic targets and clinical outcomes remains warranted to better define optimal dosing recommendations. Secondly, our simulations were limited to evaluating PTA after the first dose, rather than following multiple doses, where accumulation may occur. We prioritised evaluating the efficacious and safe use of gentamicin from the first dose, considering that (i) rapid achievement of therapeutic targets within the first hours of suspected infection are critical [29], (ii) treatment is often discontinued within 48–72 h where infection is not confirmed, or switched to targeted therapy where infection is confirmed, and (iii) clinical practice guidelines recommend therapeutic drug monitoring to ensure efficacious and safe use where treatment extends beyond 48 h. Thirdly, while simulations were limited to gentamicin administration, the observed variability in treatment response is likely applicable to other aminoglycosides given their similar pharmacokinetic profiles. Lastly, it was beyond the scope of this study to develop optimised dosing recommendations to be used internationally as this requires agreement on what the therapeutic targets should be for treating neonatal sepsis, and also consideration of individual MICs for pathogens causing neonatal sepsis which is likely to differ across neonatal units.

## 4. Materials and Methods

### 4.1. Identification of Neonatal Gentamicin Dosing Guidelines

We contacted each Australian Level 3 neonatal nursery, identified through the member list of the Australian New Zealand Neonatal Network, to obtain a copy of their gentamicin guidelines. We extracted details of demographic characteristics (i.e., GA, PNA) used to determine dose per administration and dosing interval.

### 4.2. Population Pharmacokinetic Modelling

The literature was searched to identify potentially suitable population models describing gentamicin pharmacokinetics in neonates. Optimal model selection was based on (i) the data on which the model was developed including the population of interest (GA 23–40 weeks, PNA 0–28 days), (ii) the robustness of data used for model development (the number of subjects and concentration measurements), (iii) the covariate effects included in the model, and (iv) the assessment of the predictive performance of the model (model validated by an external dataset). The selected model was coded using the mrgsolve package in R Version 4.0.5 (R Foundation for Statistical Computing, Vienna, Austria) and validated against published data.

The selected population pharmacokinetic model was used to simulate predicted gentamicin concentration–time profiles in a representative neonatal population after administration of gentamicin according to each of the identified guideline-specified dosing regimens. The in silico neonatal dataset comprised 36,960 individuals with uniform distributions of sex, GA (22–40 weeks), and PNA (1–28 days), and corresponding birth and current weight values determined using a published model [30]. Individuals with birth weight < 0.35 kg or >5 kg were excluded from the population dataset.

### 4.3. Probability of Target Attainment

Simulated concentration–time profiles were used to evaluate the ability of each guideline to meet therapeutic targets for efficacy and safety. The probability of target attainment (PTA) was taken as the proportion of patients within the population of interest that are predicted to meet the relevant efficacy/safety target.

A Cmax/MIC ratio > 10 was selected as the pharmacodynamic surrogate [11,31]. Gentamicin target peak concentrations were set at ≥5 and ≥10 mg/L (corresponding to MIC breakpoints of 0.5 and 1.0 mg/L, respectively). The peak concentrations were retrieved at 30 min post the start of infusion. Target trough concentrations were set at ≤2 and ≤1 mg/L and retrieved at the end of the corresponding dosing interval (i.e., at 24, 36, or 48 h). We also evaluated the ability of guidelines to meet combined efficacy and safety targets of a peak concentration of either ≥10 mg/L or ≥5 mg/L and a trough concentration of either ≤1 or ≤2 mg/L).

### 4.4. Statistical Analysis

Data evaluation and visual representations were performed with R (v3.1.2; R Development Core Team, Vienna, Austria).

## 5. Conclusions

This model-based simulation study demonstrates the considerable variability in the ability for existing Australian gentamicin guidelines to achieve desired therapeutic target concentrations across all neonatal age groups. The lowest likelihood of target attainment was consistently evident among extremely preterm infants, the population potentially at greatest risk from neonatal sepsis. Future research determining optimal therapeutic targets with respect to clinical outcomes appears warranted. Despite ongoing uncertainties that remain regarding optimal target concentrations, the insights provided by this study can be utilised to assist sites in initially benchmarking their guidelines nationally, while also assisting the development of new standardised national guidelines to improve clinical care and outcomes for all neonatal subgroups.

## Figures and Tables

**Table 1:** Summary of Australian neonatal unit dosing guidelines for gentamicin.

Guideline	Dose and Frequency	Infusion Time	Target Peak	Target Trough
A	PMA < 30 w: 5 mg/kg every 48 h PMA 30–35 w: 5 mg/kg every 36 h PMA ≥ 35 w: 5 mg/kg every 24 h	5 min	5–12 mg/L	<2 mg/L
B	PNA ≤ 7 d: 5 mg/kg every 36 h PNA ≥ 8 d: 5 mg/kg every 24 h	20 min	Not Stated	<1 mg/L
C	Birthweight < 1200 g: PNA ≤ 7 d: 5 mg/kg every 48 h PNA 8–30 d: 5 mg/kg every 36 h Birthweight ≥ 1200 g: PNA ≤ 7 d: 5 mg/kg every 36 h PNA > 7 d: 5 mg/kg every 24 h	30 min	Not Stated	<1 mg/L
D	GA < 33 w: PNA 0–14 d: 6 mg/kg every 48 h PNA > 14 d: 6 mg/kg every 24 h GA ≥ 33 w: PNA 0–14 d: 5 mg/kg every 24 h PNA > 14 d: 6 mg/kg every 24 h	5 min	Not Stated	<1 mg/L
E	PMA < 30 w: PNA 0–7 d: 5 mg/kg every 48 h PNA > 7 d: 5 mg/kg every 24 h PMA 30–35 w: PNA 0–7 d: 6 mg/kg every 48 h PNA > 7 d: 6 mg/kg every 24 h PMA > 35 w: PNA 0–14 d: 4.5 mg/kg every 24 h PNA > 14 d: 7 mg/kg every 24 h	10 min	>10 mg/L	<1 mg/L

Abbreviations: PMA, postmenstrual age; PNA, postnatal age; GA, gestational age.

**Table 2:** Median probability of target attainment across all gestational age groups for Australian guidelines for achieving individual and combined peak and trough target concentrations.

Therapeutic Target	PNA Group (Days)	Guideline				
		A	B	C	D	E
		Median (Min–Max)				
Peak ≥ 10 mg/L	1–7	44 (12–84)	37 (9–81)	31 (7–77)	66 (44–85)	50 (12–89)
	8–14	37 (9–83)	31 (7–77)	26 (5–72)	67 (43–83)	49 (9–86)
	15–21	39 (12–74)	32 (9–68)	26 (7–60)	77 (43–96)	77 (12–100)
	22–28	36 (11–69)	30 (8–58)	24 (6–49)	72 (39–94)	73 (11–99)
Peak ≥ 5 mg/L	1–7	100 (99–100)	100 (99–100)	100 (99–100)	100 (100–100)	100 (99–100)
	8–14	100 (99–100)	100 (99–100)	100 (99–100)	100 (99–100)	100 (99–100)
	15–21	100 (99–100)	100 (99–100)	100 (99–100)	100 (99–100)	100 (99–100)
	22–28	100 (99–100)	100 (99–100)	100 (99–100)	100 (100–100)	100 (99–100)
Trough ≤ 1 mg/L	1–7	56 (10–82)	66 (1–96)	75 (10–96)	45 (5–92)	64 (10–96)
	8–14	86 (42–97)	51 (0–91)	63 (10–90)	84 (29–99)	35 (0–94)
	15–21	97 (73–99)	80 (2–99)	87 (29–99)	66 (0–96)	66 (2–94)
	22–28	99 (91–100)	92 (7–100)	95 (58–100)	81 (3–100)	81 (8–100)
Trough ≤ 2 mg/L	1–7	96 (74–99)	98 (36–100)	99 (74–100)	93 (56–99)	98 (74–100)
	8–14	99 (95–100)	96 (22–99)	97 (72–99)	99 (87–100)	90 (23–100)
	15–21	100 (99–100)	99 (47–100)	99 (89–100)	98 (26–100)	98 (47–100)
	22–28	100 (99–100)	100 (71–100)	100 (97–100)	99 (53–100)	99 (71–100)
Peak ≥ 10 mg/L and Trough ≤ 1 mg/L	1–7	12 (0–41)	11 (0–76)	10 (0–73)	20 (0–78)	19 (0–84)
	8–14	28 (0–74)	3 (0–68)	8 (0–62)	51 (2–81)	14 (0–59)
	15–21	35 (1–73)	15 (0–67)	13 (0–59)	44 (0–92)	44 (0–93)
	22–28	32 (6–69)	23 (0–58)	18 (0–48)	54 (0–94)	54 (0–99)
Peak ≥ 10 mg/L and Trough ≤ 2 mg/L	1–7	41 (1–81)	34 (0–81)	28 (0–77)	62 (11–85)	46 (1–89)
	8–14	37 (6–82)	27 (0–76)	23 (0–71)	66 (31–82)	49 (0–79)
	15–21	39 (11–74)	31 (0–68)	25 (2–60)	75 (1–96)	75 (0–99)
	22–28	36 (10–69)	29 (0–58)	24 (4–49)	72 (5–94)	73 (0–99)
Peak ≥ 5 mg/L and Trough ≤ 1 mg/L	1–7	56 (10–82)	66 (1–96)	75 (10–96)	45 (5–92)	64 (10–96)
	8–14	86 (41–97)	51 (0–91)	63 (9–90)	84 (29–99)	35 (0–94)
	15–21	97 (73–99)	80 (1–99)	87 (28–99)	66 (0–96)	66 (2–94)
	22–28	99 (91–100)	92 (7–100)	94 (58–99)	81 (3–100)	81 (8–100)
Peak ≥ 5 mg/L and Trough ≤ 2 mg/L	1–7	96 (74–99)	98 (36–100)	99 (73–100)	93 (56–99)	98 (74–100)
	8–14	99 (95–100)	96 (22–99)	97 (71–99)	99 (87–100)	90 (23–100)
	15–21	100 (99–100)	99 (46–100)	99 (89–100)	98 (82–100)	98 (46–99)
	22–28	100 (99–100)	99 (70–100)	99 (96–100)	99 (53–100)	99 (71–100)

Abbreviations: PNA, postnatal age.

## Data Availability

Statistical code and corresponding datasets are available on request from the corresponding author: luke.grzeskowiak@flinders.edu.au.

## Supplementary Materials

The following supporting information can be downloaded at: https://www.mdpi.com/article/10.3390/antibiotics14010048/s1, Figure S1: Patient characteristics of the representative neonatal population (N = 36,772; 18,296 males, 18,476 females). Distribution of, and correlation between, gestational age, postnatal age, current weight, and birth weight. Figure S2: Median peak (Cmax) concentration (mg/L) according to individual guidelines, by gestational age and postnatal age. Figure S3: Median trough (Cmin) concentration (mg/L) according to individual guidelines, by gestational age and postnatal age.
