# Supplementary material for: A Quantitative Examination and Comparison of the Ability of Australian Gentamicin Dosing Guidelines to Achieve Target Therapeutic Concentrations in Neonates
Source: Antibiotics (Basel). 2025 Jan 8;14(1):48. doi: 10.3390/antibiotics14010048 (PMC11759765; doi:10.3390/antibiotics14010048)

Supplemental Information

A Quantitative Examination and Comparison of the Ability of Australian Gentamicin Dosing Guidelines to Achieve Target Therapeutic Concentrations in Neonates

Luke E. Grzeskowiak [1,2,3,4,*], Sheree Wynne [4] and Michael J. Stark [2,5]

[1] College of Medicine and Public Health, Flinders University, Adelaide 5042, Australia
[2] Adelaide Medical School, Robinson Research Institute, The University of Adelaide, Adelaide 5005, Australia; michael.stark@adelaide.edu.au
[3] Women and Kids Theme, South Australian Health and Medical Research Institute, Adelaide 5000, Australia
[4] SA Pharmacy, Flinders Medical Centre, SA Health, Adelaide 5042, Australia; sheree.wynne@sa.gov.au
[5] Department of Neonatal Medicine, Women's and Children's Hospital, Adelaide 5006, Australia
* Correspondence: luke.grzeskowiak@flinders.edu.au

Figure S1. Characteristics of the representative neonatal population (N=36,772; 18,296 males, 18,476 females). Distribution and correlation between, gestational age, post-natal age, current weight, and birth weight.

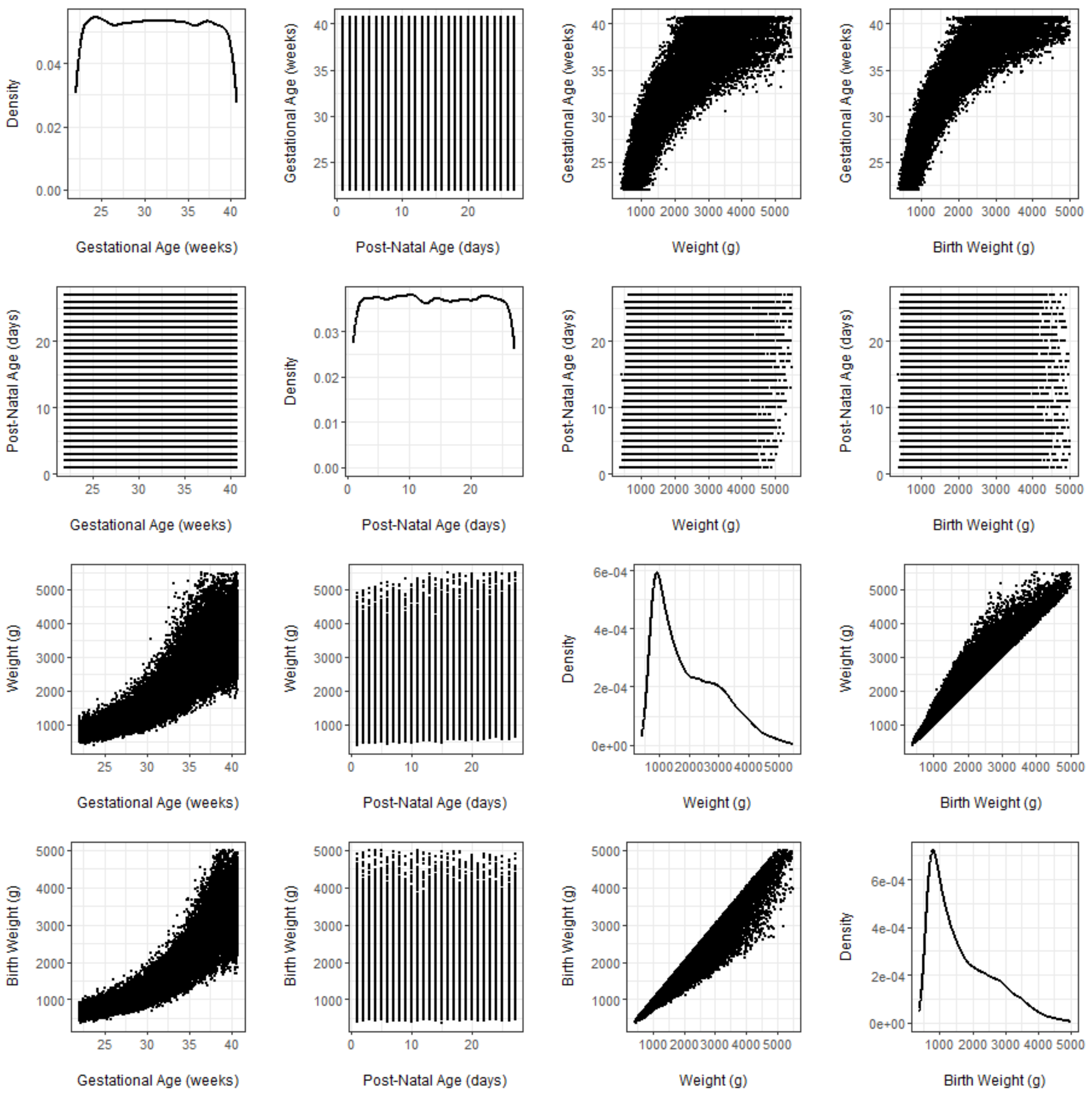

Figure S2. Median peak (Cmax) concentration (mg/L) according to individual guidelines, by gestational age and postnatal age

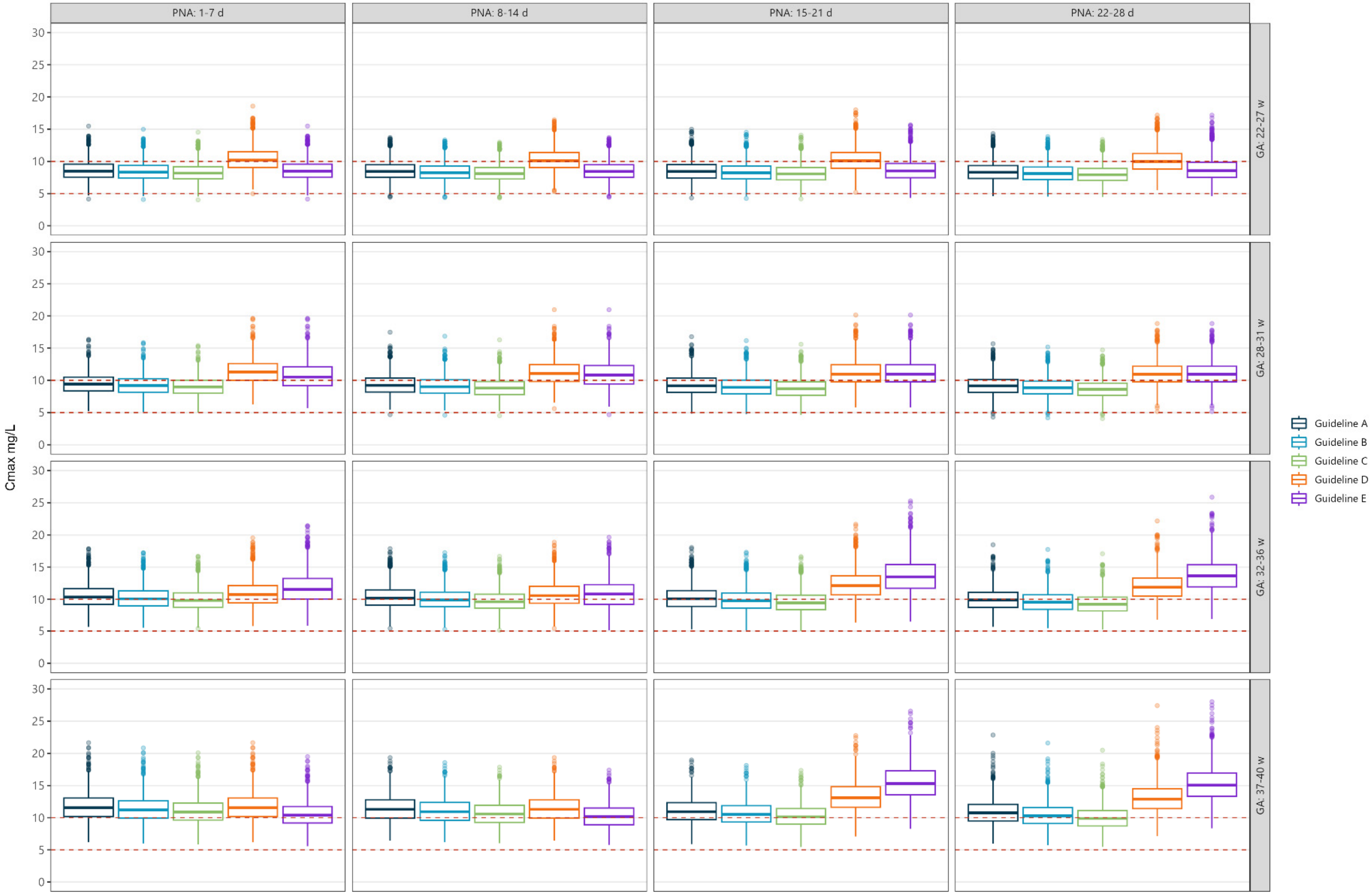

Figure S3. Median trough (Cmin) concentration (mg/L) according to individual guidelines, by gestational age and postnatal age

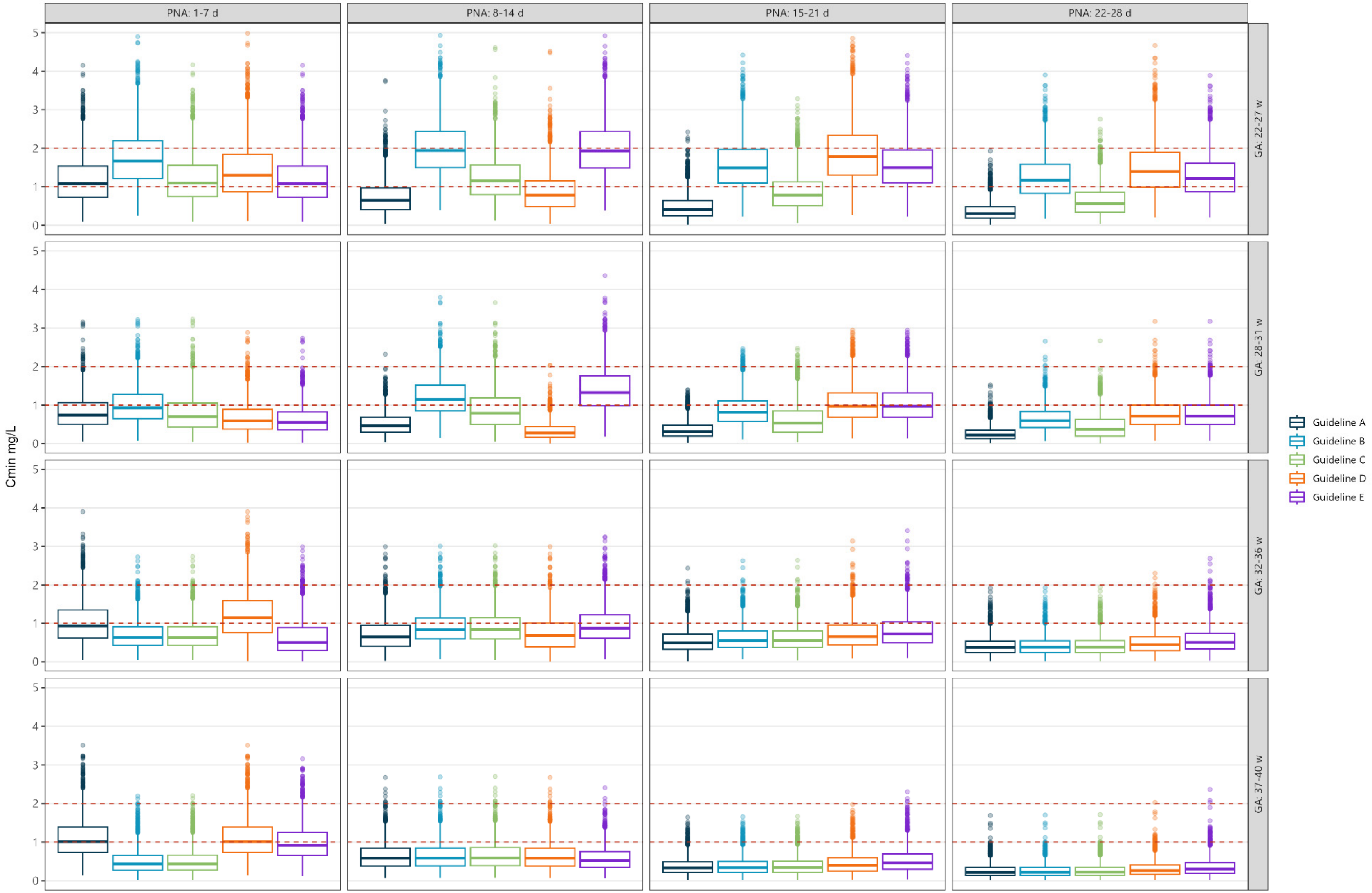

Supplement: Supplementary file 1 [file antibiotics-14-00048-s001.zip › antibiotics-3381190-supplementary.pdf]
